# Deep Brain Stimulation for Consciousness Disorders; Technical and Ethical Considerations

**DOI:** 10.1007/s12152-024-09570-5

**Published:** 2024-07-30

**Authors:** Alceste Deli, Alexander L. Green

**Affiliations:** Nuffield Department of Surgical Sciences and Nuffield Department of Clinical Neurosciences, University of Oxford, Oxford, UK

**Keywords:** Deep Brain Stimulation, Disorders of Consciousness, Neurotechnology, Neuromodulation

## Abstract

Disorders of Consciousness (DoC) result in profound functional impairment, adversely affecting the lives of a predominantly younger patient population. Currently, effective treatment options for those who have reached chronicity (prolonged symptom duration over 4 weeks) are extremely limited, with the majority of such cases facing life-long dependence on carers and a poor quality of life. Here we briefly review the current evidence on caseload, diagnostic and management options in the United Kingdom (UK), United States of America (USA) and the European Union (EU). We identify key differences as well as similarities in these approaches across respective healthcare systems, highlighting unmet needs in this population. We subsequently present past efforts and the most recent advances in the field of surgical modulation of consciousness through implantable neurostimulation systems. We examine the ethical dilemmas that such a treatment approach may pose, proposing mediating solutions and methodological adjustments to address these concerns. Overall, we argue that there is a strong case for the utilisation of deep brain stimulation (DBS) in the DoC patient cohort. This is based on both promising results of recent clinical trials as well as technological developments. We propose a revitalization of surgical neuromodulation for DoC with a multicenter, multidisciplinary approach and strict monitoring guidelines, in order to not only advance treatment options but also ensure the safeguarding of patients’ welfare and dignity.

## Introduction

Catastrophic insults to the central nervous system, impacting either arousal (wakefulness) or awareness (information processing), will lead to disorders of conscious experience in a previously healthy individual. A key pathophysiological feature of disorders of consciousness (DoC) following traumatic brain injury (TBI), haemorrhagic or ischaemic stroke, is cortical de-afferentation: disconnection between cortical and subcortical regions and disruption of cortico-cortical long range projections. This may also be the result of a neurodegenerative aetiology, but invariably results in disruption of excitatory neurotransmission to cortical areas and subsequent dysregulated arousal and awareness [1]. While both TBI-induced cell death and neurodegeneration share common pathways [2], the latter is characterised by an ever-evolving process of neuronal loss and circuit pathology [3]. Despite long-term sequelae of an initial traumatic injury, the absence of such a persistent insult might mean that a timely and efficient treatment option, geared towards restoring circuit function, could lead to long-lasting beneficial patient outcomes.

A direct method of modulating arousal-related neural circuits is the selective application of electrical pulses to the brain, through deep brain stimulation (DBS) of subcortical centers. This surgical treatment, first licenced for pharmacoresistant movement disorders, has seen an expansion of its indications in recent years [4]. A favourable risk–benefit ratio, together with the scientific validity of the proposed treatment and subsequent value enhancement of participants’ health, has been posited as being part of the basis of ethical clinical research [5]. The invasive nature and variable results of most surgical interventions for restoration of consciousness have led to a hesitancy in their systematic exploration in Western medicine and a failure of their translation into standard clinical practice. We argue that it is time to re-evaluate this approach and initiate a broader vein of controlled empirical investigation of the value of DBS applications in DoC. To this end, after a brief overview of the current case burden and diagnostic practices, we examine specific neuroethical considerations in this discourse, in the additional light of recent clinical trial evidence and technological advances in the implantable neuromodulation field.

## Current Case Load and Diagnostic Criteria: an Underserved Population?

The DoC spectrum of clinical presentations encompasses an evolving range of behaviours [6]. A key development in this field was the formal differentiation between unresponsive wakefulness syndrome (UWS, previously referred to as persistent vegetative state) and minimally conscious state (MCS), based on the fact that patients with the latter diagnosis display ‘clearly discernible, behavioural evidence’ of consciousness distinct from coma and UWS, albeit this being inconsistent in nature [7]. Where elements of language comprehension or expression are present, a diagnosis of MCS + is made (as opposed to MCS- where they are absent) [8]. Visual pursuit is the most common early sign of consciousness recovery [9], while ‘emergence’ from MCS indicates objective recovery of functional communication or object use.

In the case of ‘chronic’ or prolonged DoC (PDoC, ≥ 4 weeks from insult onset), patients may still proceed to functional recovery at a later stage. However, the rate of this is relatively low (13–30% based on a relatively recent study with a 6-week observational period [7]). Furthermore, based on data from the same trial, recovery rates are three times higher in cases who are within the first ten weeks of injury at enrolment, compared to those further removed from time of injury. These results highlight the fact that patients with PDoC represent a population with prolonged, severe disability where treatment options are scarce. This becomes even more poignant when one considers that the peak incidence of severe TBI is between 16 and 35 years of age in the USA [10]. A young adult peak is also true for European data, although a second, falls-related, peak is noted for those over 75 years of age [11].

Currently, the UK National Health Service does not collect systematic data to identify patients in PDoC, therefore there is no way of accurately identifying the number of such cases, their management and their outcomes [12]. However, based on surrogate metrics and estimates, such as functional independence measures, there may be around 150 new cases every year in the UK that fail to reach emergence after PDoC and remain in care facilities and rehabilitation centers. From the subset of ‘optimal prognosis’ achieving emergence post-DoC, 30% are discharged to long-term care in a nursing home after emergence due to prolonged disability. In terms of healthcare and other economic costs however, emergence from PDoC still generates a mean net of savings of over £436 K per patient and can effectively compensate for losses from other patient groups in the UK [12]. This fact, in addition to the potential alleviation of human suffering, further supports the importance for effective management and treatment options in the PDoC cohort.

In addition to this lack of systematic, centralised monitoring of outcomes for PDoC cases in the UK, a further heterogeneity can be noted with regards to diagnostic approaches between this country and corresponding US and EU authorities. As per the latest updated guidance from the Royal College of Physicians (2020), differential diagnosis and management in disorders of consciousness (DoC) relies upon the clinical observation of behaviours ‘*suggesting awareness of self and the environment*’ [13]. In contrast to the earlier US guidance (2018), where functional neuroimaging (fMRI) and advanced electrophysiological studies are recommended in cases of ambiguity [14], the UK guidelines do not envisage any role for these investigations in the clinical management and diagnosis of DoC. This decision is also in contrast to the updated European Academy of Neurology (EAN) guideline, which recommends an integration of EEG and fMRI studies with clinical observations, and that ‘*state of consciousness should be classified according to the highest level revealed by any of these three approaches’* [15]. Behavioural scales only provide a single snapshot captured over minutes of clinical evaluation [16], while fluctuations in state of consciousness have been linked to worse outcomes in neurocritical care populations [17]. Some hesitations in the recommendation of multimodal monitoring stem from the heterogeneity of results and the difficulty in interpretation of intentionality. For instance, fewer than half of patients with traumatic brain injury (TBI) demonstrating command-following on EEG had the same result in fMRI [18], while correlation of neuroimaging findings to motor responses in a clinical context can be especially challenging in this cohort [19].

## Surgical Neuromodulation in Disorders of Consciousness: a Viable Treatment Avenue

Despite these differences in guidelines, a common element in the management of patients with DoC, both in the UK and abroad, is the off-label use of stimulants during the rehabilitation process. Amantadine (a dopaminergic and NMDA agonist) has been shown to increase the rate of cognitive recovery in the acute phase in patients with TBI in large, multicentre trials [20, 21]. However, there is robust evidence that it offers no additional benefit in the chronic stages of DoC (seven centre, placebo-controlled, randomised clinical trial [22]). Furthermore, when Glasgow Coma Scale (GCS) outcomes were assessed, new evidence comparing the agent to modafinil (another commonly prescribed stimulant) failed to demonstrate any benefit for either drug, compared to standard-of-care rehabilitation in the U.S healthcare system [23]. Stimulant efficacy may depend on factors such as the half-life of each agent and time of dosing with respect to behavioural fluctuations – therefore personalisation of treatment can be limited and depend on carer attention. This brings into question whether responsive treatments, taking into account immediate changes of patient physiology and behaviour, might be more effective and ethically advantageous –provided they do not expose the patient to disproportionate associated risks.

An argument against the use of DBS is that the invasiveness of the technique carries significant risks, such as intracerebral haemorrhage, subsequent disability and even death. With regards to bleeding as a complication of the procedure, the incidence has been estimated to be 5% in a comprehensive review of available published data [24], however risk varies with age and associated comorbidities. Although, as we previously discussed, the PDoC cohort may center around a relatively younger population, a personalised risk assessment would ensure that all relevant factors are accounted for, before proceeding to a decision for surgical management. Furthermore, there is empirical evidence that DBS can beneficially alter symptoms in PDoC, where pharmacological interventions are of limited benefit. In the context of DoC after 6 months post-insult, a number of US and EU studies focused on DBS of the centrolateral and centromedian-parafascicularis nuclei of the thalamus, spanning over thirty years [25–27].

The scientific premise behind this approach lies in the fundamental role that thalamocortical neuronal loops play in both information representation [28] and arousal maintenance, via their link to the reticular activating system [29]. Recent experimental evidence in primates has showcased that thalamic DBS can restore both of these components of conscious awareness [30], further lending weight to this choice of targeting –although targeting in one surgical center also involved the globus pallidum [31]. Despite behavioural and electrophysiological changes, suggestive of a positive impact on conscious cognition, most of these studies suffer from excessive heterogeneity at baseline within small cohorts and are difficult to generalise [32]. In contrast, a recent randomised feasibility trial of thalamic DBS in a very specific population showed that it is possible to impart substantial changes, directly resulting in improved quality of life in the PDoC population [33]. This study focused on post-emergence PDoC cases who still suffered from cognitive sequelae, resulting in impairment of day-to-day functioning. This suggests that it is possible to reverse maladaptive circuit changes, rescue neuronal activity associated with arousal and awareness and achieve positive outcomes, even over a decade after the original traumatic insult. Therefore, the case for improvement of level of functioning in the PDoC cohort with DBS exists for many years following initial patient presentation.

A focus on TBI survivors who may be residing in the community, yet still require levels of care, also ensures that DBS will not produce a highly disabled state with increased awareness. Such an occurrence could be considered futile or even highly detrimental to patient experience [34]. To avoid such adverse outcomes, careful patient selection, taking into consideration dormant potential of achieving independence, is key. One therefore may wish to avoid performing DBS in vegetative state where there is extensive structural damage of circuits responsible for voluntary behavioural control. Additionally, the time window of intervention needs to be carefully considered so that the risk of DBS-induced adverse events is weighed against the potential for spontaneous emergence. An uncomplicated implant when there is small potential for emergence may just speed recovery up; a serious adverse event at an earlier timepoint may not be equally justified. However, even if the argument of futility can be overcome, there are specific ethical concerns that require further, careful consideration.

## DBS in DoC: Implications for Self-Perception, Mental Privacy and Patient Autonomy

Increasing excitatory drive through DBS may result in various emerging behaviours. Disinhibition, following dopaminergic circuit modulation, is one of the reasons behind preoperative neuropsychological screening and postoperative monitoring in DBS for movement disorders [35]. The neuroethical discourse on personality changes and alterations of perceptions of ‘self’ as sequelae of DBS is therefore understandable [36]. A critical review however of both ethical arguments and empirical evidence on the subject revealed that the purported alterations are disproportionately represented, compared to both the likelihood of post-operative changes and their nature [37]. Such effects can also occur after treatment of other neurosurgical pathologies (notably brain tumours). However, discouraging treatment which can salvage life and functioning is unwarranted on the basis of this risk, even for benign space-occupying lesions [38].

Furthermore, ‘inherent’ personality traits (versus ‘machine-imposed’ ones) are not conducive to wellbeing when highly pathological. To argue that personality should be unquestionably unalterable would therefore inevitably lead to suffering where potentially reversible, pharmacological approaches fail. This sentiment is mirrored by the FDA’s decision to grant humanitarian device exemption (HDE) for treatment of obsessive–compulsive disorder with DBS in 2009, followed by multiple trials showcasing significant improvements in overall function [39]. In the case of DoC, affective alterations (which could theoretically arise with DBS adjacent to midline serotoninergic structures [40]) could actually be beneficial in encouraging engagement with carers and motivation for recovery.

The introduction of implantable devices as an additional locus of behavioural control is however a risk to self-determination, when not performed in an appropriately regulated framework. Access to neural data through brain-computer interfaces has led to a burgeoning discourse on the need for legal protection of mental privacy [41]. Although such a right could be derived from the existing jurisprudence within the European Court of Human Rights (ECHR) [42], related clinical trials may be compromised in environments where there is rampant political autocracy, resulting in a disrespect of personal liberties. In the very specific concept of DBS in DoC, there is the risk for imposed control of arousal and awareness, as a discrete weaponised technology. Where consciousness can be selectively enhanced or dampened through DBS outside of a specific treatment indication, free will is clearly compromised [43]. Although timely condemnatory reactions of such acts of atrocity could limit further attempts, morally unacceptable events will still damage public reception of potentially life-changing treatment options [44].

The globalization of trials of DBS in DoC, involving countries where legal lacunae surrounding consenting practices exist, is an adjacent issue [45]. When consciousness is impaired, therefore patients cannot directly consent to treatment, regulatory codes such as the Code for Federal Regulations (CFR, USA) and the Declaration of Helsinki clearly indicate that a legally authorized representative must be involved, while any experimental components must be clearly declared [46, 47]. Even under such conditions, respecting a patient’s prior wishes and beliefs surrounding the management of altered states of consciousness can be challenging where there is an absence of advanced directives. If citizen inequalities exist due to gender, creed or any other characteristic, such nuanced discussions can be practically impossible. Harmonisation with global humanitarian judiciary considerations in therefore imperative in order to ensure equitable and fair access to emerging treatments, while preserving patient dignity and autonomy [48]. Regardless of environment, a multidisciplinary approach to both recruitment and disclosure of treatment effects is vital, with involvement of advocates, carers and the trial research and clinical team.

Patient autonomy can however be reinforced through novel features of implantable technologies. In movement disorders, where neural activity has been linked to specific disease states, embedded detectors of such ‘biomarkers’ have been proposed for incorporation in the device design [49]. A transition from an ‘open-loop’, physician-led approach to a responsive ‘closed-loop’ method of adaptive stimulation, directed by the patient’s own physiology and behaviour, takes into account events that occur outside the hospital [50, 51]. At present, such approaches are clinically approved for device systems used in movement disorders –although experimentally proposed for novel DBS indications such as pain [52] and depression [53]. Given the inherent difficulties in managing a PDoC cohort, such as mobility impairments, nursing/care home domicile and related difficulties in accessing clinical care, a closed-loop option, whose titration could be based on quantifiable neurophysiological markers, might facilitate management. However, difficulties in communication, endemic in this patient population, would require very close monitoring of any automated approach to neurostimulation, to ensure that side effects are properly accounted for and timely recognised.

## Conclusions and Future Directions

Despite a lengthy past of variable results, an inherently invasive nature and a highly vulnerable clinical population, neurosurgical options for the management of disorders of consciousness (DoC) should not be dismissed on either ethical or clinical efficacy grounds. We suggest that, in light of both recent updates with regards to the feasibility and effectiveness of trials for such treatment options, as well as technological advances in the field of implantable devices, the undertaking of further efforts in the field is more than morally justified.

Such efforts could also involve alternative surgical targets for arousal neuromodulation, thereby further expanding the pool of patient candidates to those where there is evidence of damage in thalamocortical connections, making stimulation of the thalamus less likely to work. In addition to their relay to intralaminar thalamic nuclei, first theorised to mediate cortical synchronisation in cats by Moruzzi and Magoun [54], brainstem and basal forebrain arousal centers project directly to cortex via extrathalamic pathways [55, 56]. Brainstem targeting has been successfully performed in humans in EU and US healthcare systems outside a DoC context, with targets including the locus coeruleus, pedunculopontine nucleus (PPN) and parabrachial complex (PBC) [57]. One notable exception are early DBS trials in Japan, where both thalamic and ‘median reticular formation’ targeting was performed for DoC –unfortunately without comparisons between thalamic and extrathalamic groups [58]. Novel work in our centre focuses on the utilisation of stimulation delivered in these regions for the amelioration of arousal pathology, concomitant to neurodegeneration in movement disorders where DBS is utilised as a treatment [59]. This follows earlier findings that brainstem stimulation can be utilised to alter sleep depth in humans, with discrete effects on both electrophysiology and behaviour when delivered during slow wave sleep [60].

No matter the DBS target and surgical approach however, further harmonisation of diagnostic and management standards in DoC is required, together with adherence to a strict ethical approach to trial structure and patient monitoring. Large multicentre clinical trials, involving an array of specialists with different types of expertise and carried out in an appropriately regulated environment are necessary, in order for a substantial change in the field to take place. The DBS field is no stranger to stunted growth due to ethical controversies. Investigations of subcortical electrical stimulation were initially carried out in the 1950s without regulatory approval [61, 62], while mounting ethical concerns led to an abandonment of such interventions in mood disorders. A tentative revisiting of this question came not only over half a century after its original ‘trial’, but also decades after FDA approval for DBS (granted in 1997, following robust proofs of efficacy in tremor control ten years earlier) [63]. The neuromodulation field should proceed with caution in DoC, since unfortunately no system is fool-proof: individual awareness and responsibility are of paramount importance.
